# *QuickStats:* Percentage of Persons of All Ages Who Delayed or Did Not Receive Medical Care During the Preceding Year Because of Cost, by U.S. Census Region of Residence* — National Health Interview Survey, 2015^†^

**DOI:** 10.15585/mmwr.mm6604a9

**Published:** 2017-02-03

**Figure:**
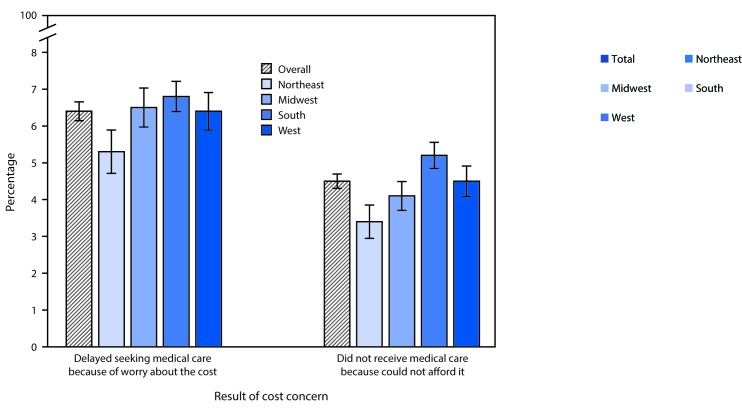
In 2015, approximately 6% of persons of all ages (20.1 million) in the United States delayed medical care during the preceding year because of worry about the cost, and 4.5% (14.2 million) did not receive needed medical care because they could not afford it. Persons living in the Northeast were significantly less likely than persons living in the Midwest, South, or West to delay or not receive needed medical care. Persons living in the South were significantly more likely to not receive needed medical care than those in the Northeast, Midwest, or West.

Source: National Health Interview Survey, 2015. https://www.cdc.gov/nchs/nhis.htm.

